# Remote Physical Frailty Monitoring–The Application of Deep Learning-Based Image Processing in Tele-Health

**DOI:** 10.1109/access.2020.3042451

**Published:** 2020-12-04

**Authors:** MOHSEN ZAHIRI, CHANGHONG WANG, MANUEL GARDEA, HUNG NGUYEN, MOHAMMAD SHAHBAZI, AMIR SHARAFKHANEH, ILSE TORRES RUIZ, CHRISTINA K. NGUYEN, MONTHAPORN S. BRYANT, BIJAN NAJAFI

**Affiliations:** 1 Michael E. DeBakey Department of Surgery, Baylor College of Medicine, Houston, TX 77030, USA; 2 Telehealth Cardio-Pulmonary Rehabilitation Program, Medical Care Line, Michael E. DeBakey VA Medical Center, Houston, TX 77030, USA; 3 Department of Physical Medicine and Rehabilitation, Baylor College of Medicine, Houston, TX 77030, USA; 4 Department of Medicine, Section of Pulmonary, Critical Care and Sleep Medicine, Baylor College of Medicine, Houston, TX 77030, USA

**Keywords:** Physical frailty, remote patient monitoring, telemedicine, mobile health, chronic obstructive pulmonary disease, digital health, deep learning

## Abstract

Remote screening physical frailty (PF) may assist in triaging patients with chronic obstructive pulmonary disease (COPD) who are in clinical priorities to visit a clinical center for preventive care. Conventional PF assessment tools have however limited feasibility for remote patient monitoring applications. To improve the safety of PF assessment, we previously developed and validated a quick and safe PF screening tool called Frailty Meter (FM). FM works by quantifying weakness, slowness, rigidity, and exhaustion during a 20-second repetitive elbow flexion/extension task using a wrist-worn sensor and generates a frailty index (FI) ranging from zero to one; higher values indicate progressively greater severity of frailty. However, the use of wrist-sensor limits its applications in telemedicine and remote patient monitoring. In this study, we developed a sensor-less FM based on deep learning-based image processing, which can be easily integrated into mobile health and enables remote assessment of physical frailty. The sensor-less FM extracts kinematic features of the forearm motion from the video of 20-second elbow flexion and extension recorded by a tablet camera, and then calculates frailty phenotypes and FI. To test the validity of sensor-less FM, 11 COPD patients admitted to a Telehealth pulmonary rehabilitation clinic and 10 healthy young volunteers (controls) were recruited. All participants completed the test indicating high feasibility. Strong correlations (0.72 *<* r *<* 0.99) were observed between the sensor-based FM and sensor-less FM to extract all frailty phenotypes and FI. After adjusting with age and body mass index(BMI), sensor-less FM enables distinguishing COPD group from controls (p*<*0.050) with the largest effect sizes observed for weakness (Cohen’s effect size d=2.24), frailty index (d=1.70), and slowness (d=1.70). These pilot findings suggest feasibility and proof of concept validity of this sensor-less FM toward remote assessment of PF in COPD patients.

## I. INTRODUCTION

Frailty is used to identify older adults with low physiological reserves and vulnerability to illness and high risk of disability, institutionalization, and death [1], [2]. Frailty syndrome is common (above 56%) in individuals with chronic obstructive pulmonary disease (COPD) [3], [4], and the frail patients tended to have a greater number of disabilities and a higher risk of mortality [5]. Therefore, frailty screening may help identifying those patients who need to receive urgent treatment, physical therapy, or pulmonary rehabilitation for preventing adverse outcomes or delaying progression toward frailty.

Frailty is often characterized by assessing physical fitness, called physical frailty (PF). Multiple tools have been developed to objectively determine PF [6], [7]. Fried frailty phenotype has been the gold-standard for clinical assessment of PF [8]. This method determines PF based on presence or absent of five phenotypes including exhaustion, inactivity, shrinking, slowness, and weakness. The first three phenotypes are subjectively assessed with surveys, whereas the last two are objectively measured with a grip force and a4.5 meters walk test. The administration of this test, specifically the walking test, is challenging in patients with limited mobility including COPD patients, who often have high risk of falling and may need to be connected to a ventilator machine. On the other hand, the lack of ability to walk does not necessarily indicate physical frailty. In addition, incomplete phenotype assessment, compromises the predictive power of the tool [9], [10]. Furthermore, these tests must be performed in clinic environments under supervision of professionals. This limitation challenges their practicality for long-term tracking of PF conditions. Particularly during public health emergencies (e.g. Covid-19 [11]), individuals with COPD are discouraged to visit clinical centers due to drastic containment and mitigation measures, further limiting the effectiveness of in-person PF assessments. Therefore, a new assessment method, which can obtain objective metrics associated with PF safely and remotely, are desperately needed.

Our team has developed and validated a quick frailty meter (sensor-based FM) tool based on a wrist-worn sensor [12]. The device enables the quantifying of PF by measuring slowness, weakness, exhaustion, and rigidity phenotypes during 20-second repetitive elbow flexion and extension test [12]. 20-second was chosen according to our prior study [13] in which 20-second repetitive elbow flexion-extension exercise was showed to be long enough to capture alterations in elbow angular velocity due to the presence of exhaustion phenotype (based on Fried Frailty Exhaustion phenotype [14]) but not too long to observe noticeable alteration in those without presence of exhaustion phenotype. Using a linear regression modeling including bootstrap with recursive feature elimination technique, the measured phenotypes are mapped into a continuous scale ranging from 0 to 1, called frailty index (FI) [12]. The sensor-based FM was validated against the Fried frailty Phenotypes Criteria [14], in a sample of 117 community dwelling older adults [13], in which 100% and 87% sensitivity were achieved to identify, respectively, frail and pre-frail cases with specificity greater than 95%. In another study [15], the sensor-based FI showed a significant agreement (r = 0.72, p *<* 0.0001) with a clinically validated trauma specific FI [16], [17] in a cohort of 101 bedbound geriatric patients with 78% sensitivity and 82% specificity to distinguish cases with frailty from non-frail individuals. In a subsequent study [18], in which the same cohort followed up to 60 days post hospital discharge, baseline sensor-based FM enables predicting those patients with and without unfavorable discharge disposition, 30-day readmission, 60-day readmission, and 30-day prospective falls. In another study [19], it was demonstrated that dual-task FM (20-second repetitive elbow flexion-extension while counting backward) enables distinguishing between older adults with and without cognitive impairment in a sample of 67 community dwelling older adults. Sensor-based FM has also been used in several clinical studies including prediction of adverse events post vascular surgery, early-stage Alzheimer’s screening, and prediction of adverse events among COPD patients [20]–[24]. Unlike the traditional PF phenotype centered on gait, the FM-based test is safer for frail older adults with impaired mobility and a high risk of falling, as the test can be administered while the subject is in a sitting or lying position. The test is in particular advantageous for COPD patients who may need to be connected to a ventilator machine and thus have difficulty performing gait test [25].

However, sensor-based FM still requires professional device operation, which may not be friendly to non tech savvy users. As a result, its application is not suitable for remote patient monitoring and telemedicine applications. To address this gap, in this study we developed a sensor-less frailty assessment tool (sensor-less FM) based on deep learning and image-processing technologies, which can be easily integrated into a tablet (mobile health) and enables remote assessment of PF without extra cost. We hypothesize that the sensor-less FM has a high agreement with the sensor-based FM to assess PF in COPD patients. Additionally, we compare PF phenotypes and index between COPD patients and young healthy people using the sensor-less FM.

## II. METHOD

**FIGURE 1.**
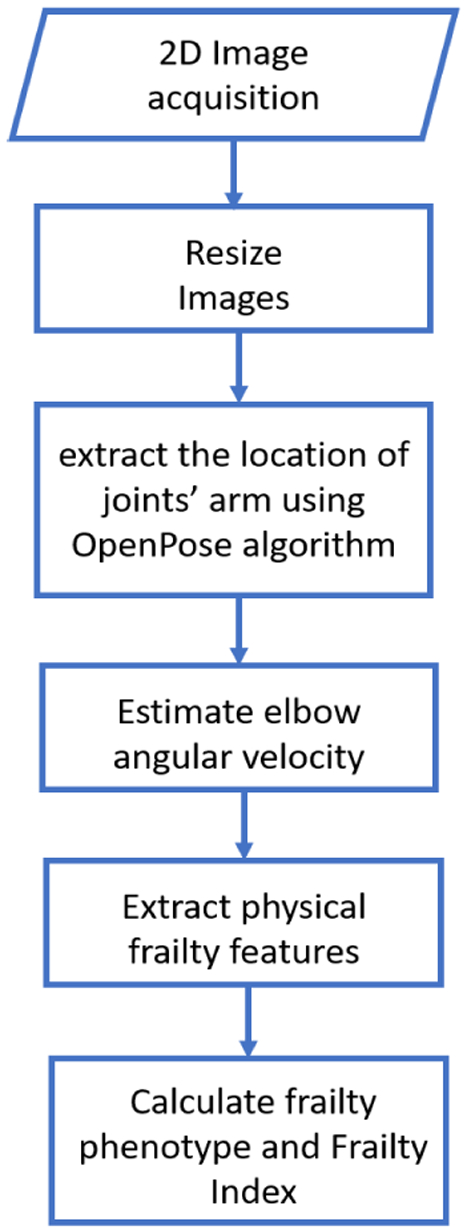
Flow chart of the algorithm to calculate physical frailty phenotypes and frailty index from the 2D video of 20-second repetitive elbow flexion and extension exercise.

Figure 1 illustrates the flowchart of the algorithm to estimate physical frailty and frailty phenotypes from a 2D video captured using a standard RGB smartphone/tablet camera. The algorithm first uses deep learning to estimate positions of wrist and elbow joints from each frame of the captured 2-D video using the approach suggested by Cao et al, called “OpenPose” algorithm [26]. In summary, OpenPose algorithm uses convolutional networks that enables predicting the location of a joint of interest (see section A. image processing section). The advantage of this algorithm is the use of 2D video to track the position of a body joint instead of 3D video which is required in the conventional motion tracking systems. Based on changing the wrist and elbow positions between two consecutive frames, the angular velocity of the forearm around elbow joint is calculated. Then, the features of interest (e.g. number of flexion and extension, reduction in angular velocity over time, elbow flexion time, elbow extension time) are extracted from the angular velocity as described in details in the following (see section B: Feature Extraction). At the end, physical frailty phenotypes (i.e. slowness, weakness, exhaustion, and rigidity) and frailty index is obtained from the features.

### A. IMAGE PROCESSING – OpenPose ALGORITHM

**FIGURE 2.**
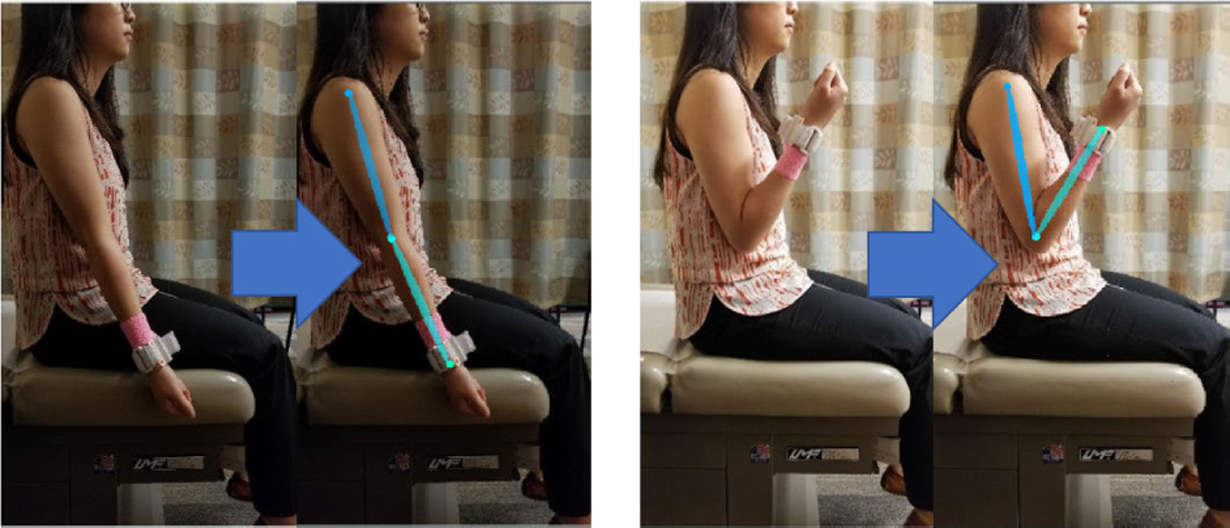
Estimation of wrist and elbow joints locations using the “OpenPose” algorithm in two example frames.

A 2D video, with the speed of 30 frames per second, was captured from a tablet camera (Samsung Galaxy Tab, Seoul, South Korea) for the 20-second rapid repetitive elbow flexion and extension test. In each frame of the video, the positions of the joints of the arm are achieved by encoding the arm pose of subjects using OpenPose algorithm as a pre-trained deep learning method [26], [27]. This multi-stage convolutional architecture iteratively predicts 2D anatomical joints of the arm for each person in the frame (Figure 2). The model takes a color image as an input and outputs an array of matrices including a confidence maps and affinity fields. In the first step, the first 10 layers of the VGG-19 net is used to produce the feature maps from the input image. The second step of the model consists of a 2-branch multi-stage CNN to predict a set of 2D confidence maps and affinity fields. The confidence map is a grayscale image which shows the location of key point (e.g. wrist and elbow) in the image and affinity fields encodes the degree of association between key points. The angular velocity of the elbow can be extracted based on the positions of the wrist and the elbow in the sequence of frames.

### B. FEATURE EXTRACTION

**FIGURE 3.**
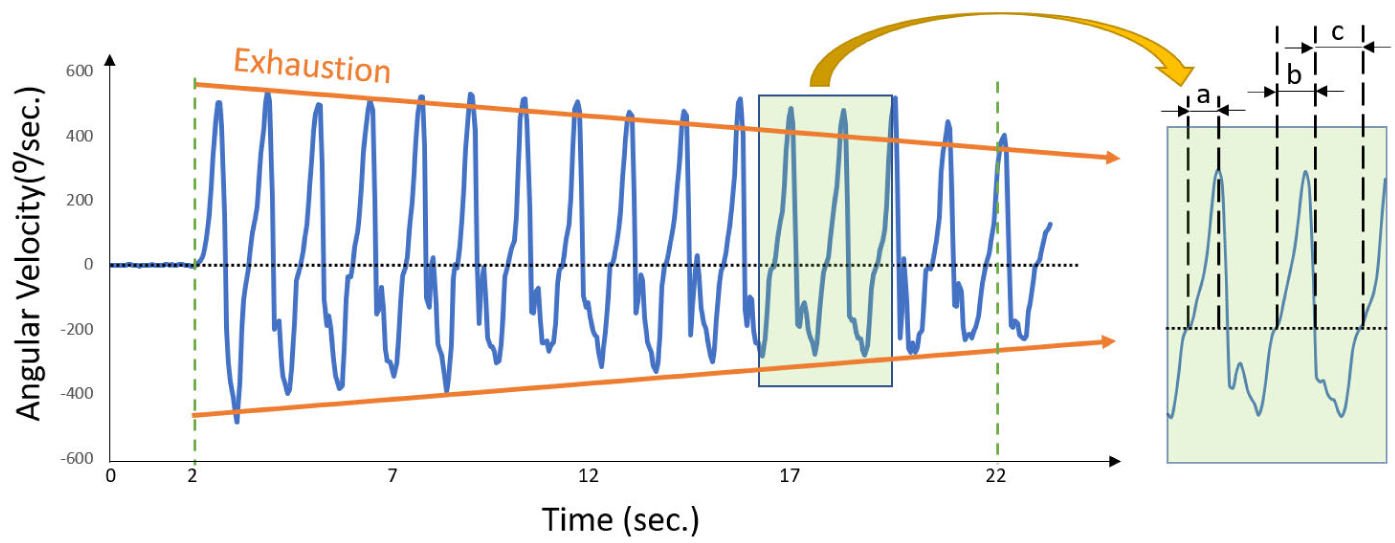
A typical pattern of angular velocity recorded by FM. Reduction in angular velocity, rise time(a), and power over the 20-second period of elbow flexion and extension are markers of exhaustion, slowness, and weakness. Flexion time (b) and extension time (c) used to calculate Frailty Index.

The features of interest from the 20-second elbow repetitive flexion-extension test were extracted according to Lee et al study [12] in which sensor-derived digital biomarkers related to physical frailty phenotypes and measurable from elbow angular velocity (Figure 3) during 20-second repetitive elbow flextion-extension test were defined. Table 1 summarizes these features and their definition. In summary, we used a zero-crossing method and peak detection algorithm to distinguish each extension/flexion period from the 20-second angular velocity signal [12]. Then 20 features were extracted to determine slowness, weakness, rigidity, exhaustion, and unsteadiness frailty phenotypes. Five features were used to determine slowness including angular velocity range, rise time (Fig 3-a), fall time, extension time (Fig 3-b), flexion time (Fig 3-c), and number of extensions/flexions. The weakness was characterized by estimating the product of range of angular velocity and range of angular acceleration. Rigidity was quantified by estimating the elbow flexion/extension range of motion. Elbow rotational angle was calculated using quaternion and Kalman filters as described in our previous study [28]. Exhaustion was quantified by the magnitude of change in features of interest over a 20-second test including a decline in ‘elbow speed of rotation’, decline in ‘power’, increase in ‘flexion/extension’ time, and increase in ‘rise time’. To determine change over 20-second, the differences between the first and last 10 seconds of features of interest were calculated. To quantify unsteadiness, we calculated the coefficient of variation (CV) of five features as summarized in Table 1.

In our previous study and in a cohort of 100 geriatric inpatients, we identified the optimized combination of features to determine frailty index using a regression model, bootstrap with 2000 iteration, and recursive feature elimination technique [12]. The estimated frailty index based on the optimized linear regression model is as follow:

(1) $${\text{FI}}_{\text{est}}=-1.7357\times 10^{-3}{\text{Ph}}_{1}-1.2026\times 10^{-3}{\text{Ph}}_{2}+0.36848\times 10^{-3}{\text{Ph}}_{3}-0.49396{\text{Ph}}_{4}+0.48974{\text{Ph}}_{5}+0.24495,$$

where Ph represents the selected features of interest as: Ph_1_ – range of motion; Ph_2_ – percentage of decline in power; Ph_3_ – flexion time; Ph_4_ –flexion time variability; and Ph_5_ –extension time variability. Frailty index ranges from 0 to 1; higher values indicate progressively greater severity of physical frailty.

## III. TEST

For the setting of the test, the subject wore a wrist-worn sensor (sensor-based FM, a tri-axial gyroscope, sample frequency = 100 Hz, BioSensics LLC, MA, USA) as a bench mark. The participant was asked to repetitively flex and extend their dominant elbow to full flexion and extension as quickly as possible for 20 seconds. While the test was performed, the subjects were recorded on their sagittal plane by a tablet camera (Samsung Galaxy Tab, Seoul, South Korea) ensuring arm flexion and extension remained in the video (sensor-less FM).

### A. DATA ACQUISITION

We recruited 11 patients (age: 67.8±10.7 years old, BMI:32.2±25.1 kg/m^2^, 82% frail or pre-frail according to Fried Frailty Criteria) with Chronic Obstructive Pulmonary Disease (COPD) from the Telehealth Pulmonary Rehabilitation Clinic at Michael E. DeBakey Veterans Affairs Medical Center – Houston, Texas, USA. We also recruited 10 healthy young subjects (age=29.6±6.7 old, BMI:28.7±6.7kg/m^2^), from the staff and students at the Baylor College of Medicine. Common eligibility criteria include their ability to provide written informed consent and ability to do 20-second elbow flexion and extension. Participants were excluded from the study if they were non-ambulatory had neurological conditions affecting upper extremity function (recent stroke, Parkinson disease, Huntington disease, etc.); or were unwilling to participate. All participants signed a consent form for this study. This study was approved by the Institutional Review Board of the Baylor College of Medicine and the Michael E.

DeBakey Veterans Affairs Medical Center (Houston, TX, USA).

### B. STATISTICAL ANALYSIS

To assess the agreement between the sensor-based FM and the proposed sensor-less FM, we used Altman and Bland (B&A) plot on these two quantitative measurements [29]. This plot examines the association between the difference and the mean of the paired measurements. The Y axis presents the difference between the paired measurements and the X axis shows the mean of the paired measurements. The upper and lower 95% limits are also presented to show the limit of agreement (mean of difference ± 1.96 × SD of difference).

We evaluated the correlation of frailty phenotypes between the sensor-less and sensor-based FM by using the Pearson correlation coefficients [30]. For comparison between two methods, in addition to the estimated frailty index, we selected the features shown to be independent predictors of frailty index based on prior studies [12]–[15]. Values ranging from 0.40–0.59 indicates moderate correlation, from0.6–0.79 indicates strong correlation, and from 0.8–1.0 indicates very strong correlation [31]. Univariate general linear model was used to compare the frailty parameters between COPD subjects and healthy control group. Results were adjusted by age and BMI. The effect size for discriminating between groups was estimated using Cohen’s *d* effect size and represented as *d* in the Results section. Values were defined as small (0.20–0.49), medium (0.50–0.79), large (0.80–1.29), and very large (above 1.30) [32]. Values less than0.20 were classified as having no noticeable effect [32]. In our analyses, statistical significance was accepted when p *<* 0.05.

## IV. RESULTS

### A. ACCURACY OF IMAGE BASED SYSTEM TO ESTIMATE THE FRAILTY PHENOTYPE AND FRAILTY INDEX

**FIGURE 4.**
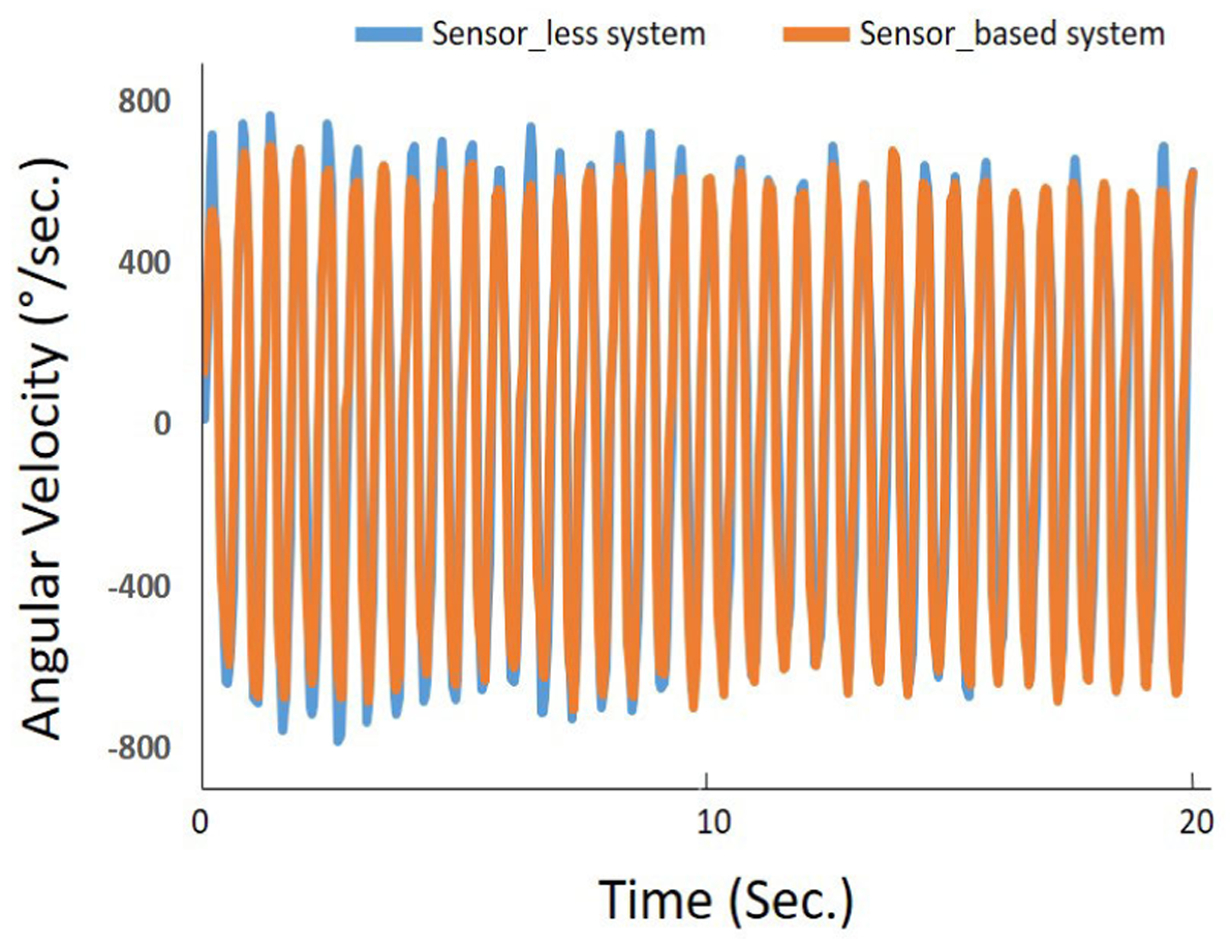
Elbow angular velocity. The orange signal shows the sensor-based signal as our gold standard. The Blue signal demonstrates the output of the sensor-less FM to define the position of forearm in the frames of the video.

All participants completed the test indicating high feasibility. Figure 4 demonstrates the angular velocity extracted from the forearm movement for both sensor-based and sensor-less FMs in a typical COPD subject.

There was strong to very strong correlations between the sensor-less FM and the sensor-based FM to estimate the frailty phenotypes (Table 2 ). Very strong correlation with statistical significance was observed for rise time (slowness phenotype), power (weakness phenotype), and elbow flexion time. Strong correlation with statistical significance was observed for range of rotation (rigidity phenotype), decline in speed, decline in power (exhaustion phenotype), and frailty index.

**FIGURE 5.**
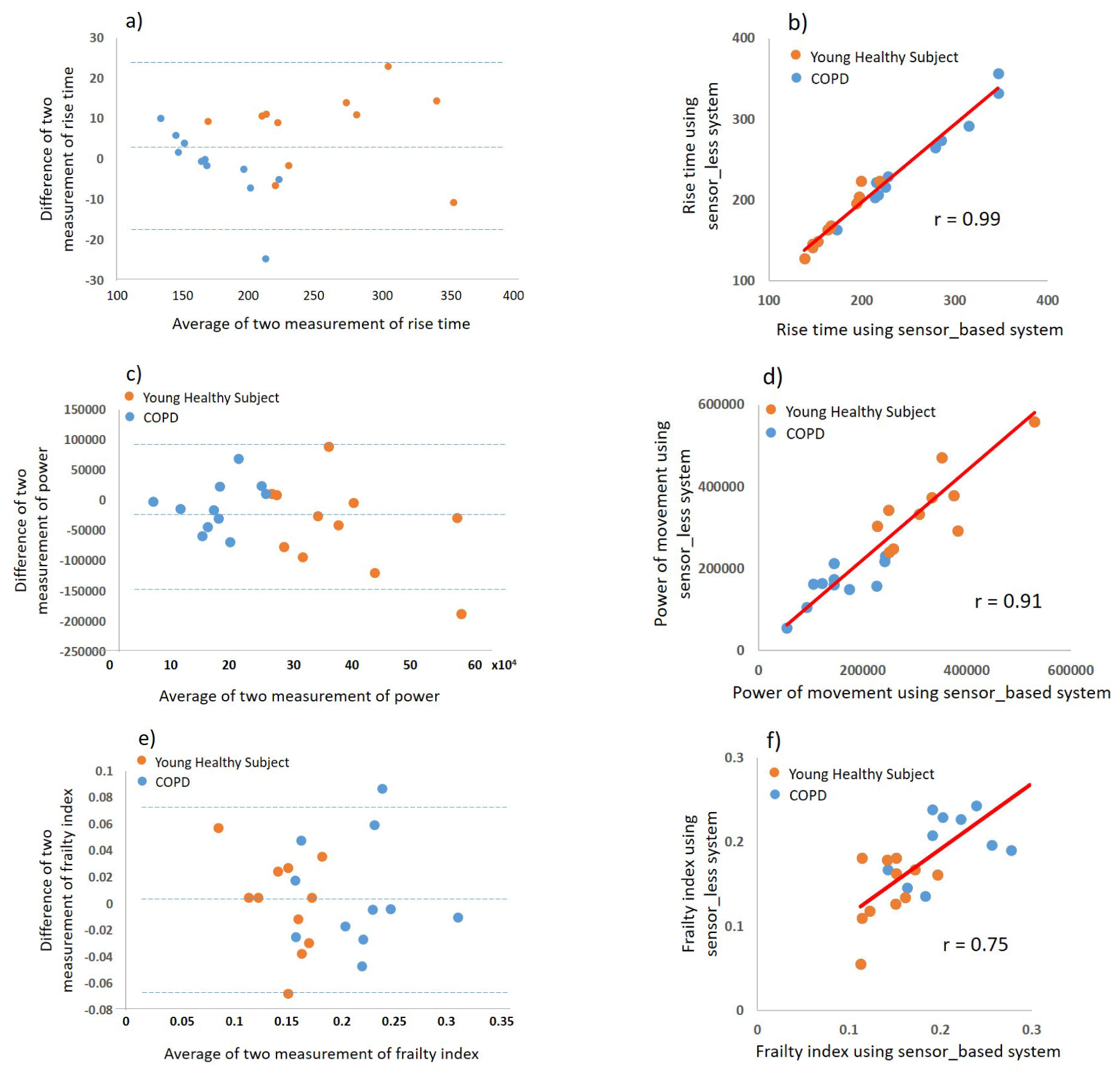
Bland-Altman plots showing the agreement between the sensor-based FM and the sensor-less FM for (a) rise time, (c) power, and (e) frailty index. Scatterplots between the sensor-based FM and the sensor-less FM for (b) rise time, (d) power, and (f) frailty index.

The Bland-Altman plots for all phenotypes show a high precision and low bias for estimating frailty phenotypes using the sensor-less FM compared to the sensor-based FM (Figure 5). Figure 5 (e) and (f) suggest a strong agreement between the estimated frailty index from the proposed sensor-less FM compared to the sensor-based FM.

### B. FEASIBILITY OF SENSOR-LESS SYSTEM TO SEPARATE THE COPD GROUP FROM CONTROL GROUP

Table 3 summarized the differences between group for key phenotypes including rise time (phenotype slowness, Unit: s), power (phenotype weakness, Unit deg^2^/s^3^ × 100,000), rigidity (elbow range of motion, deg), change in power (exhaustion, Unit: %, negative sign: decline; positive sign: increase), and frailty index. All phenotypes except rigidity and exhaustion were significantly different between COPD group and control group (p*<*0.007). The largest effect sizes were observed for weakness (d=2.41), frailty index (d=1.70), and slowness (d=1.62).

## V. DISCUSSION

This study proposes a telehealth platform to assess physical frailty based on a tablet with a camera module. The platform is practical for remote tracking of physical frailty status, which in turn may assist with triaging high risk patients and timely intervention to reduce frailty-related health deterioration among the COPD patient population. The key advantages of the proposed sensor-less FM are ease of use (20 seconds to complete the test with no need of sensor attachment), low cost (using a mobile app), and high safety (no need to use a walking test and the test can be administered while sitting or lying down on a bed).

From Figure 4, we can observe a good agreement for angular velocity signals outputted from the sensor-less and the sensor-based FMs. The high agreements observed between sensor-based and sensor-less derived features correlation, suggest that sensor-less FM is as accurate as sensor-based FM for determining frailty phenotypes of interest. As a result, the derived frailty index based on these sensor-less kinematic parameters has a good agreement with the sensor-based FM. The B&A plots (Figure 5) further support good agreements for the frailty phenotypes and the frailty index between the sensor-less FM and the sensor-based FM. In summary, these results indicate a good accuracy of the proposed sensor-less FM to assess physical frailty.

As expected, results suggest COPD patients were significantly more physically frail than the healthy control subjects based on the frailty phenotypes and frailty index obtained using the sensor-less FM. The results are in line with the other known studies [4], [5], [3]. This finding indirectly verifies the effectiveness of the sensor-less FM to determine physical frailty. In addition, our study suggests that the most pronounced phenotype affected by COPD is weakness followed by slowness.

This study has two main limitations. First, this study examined the sensor-less FM in the clinic environment under supervision of a research coordinator. This testing condition ensures the consistency across different subjects and excludes as much interference from human factors as possible. For example, in our trial, the subject’s sagittal plane is always approximately perpendicular to the sight line of the camera, and all forearm movements are completely captured in the view of the camera based on real-time observation of the research coordinator. However, for the remote application, various factors including home environment (e.g., light), ability to follow instruction to examine sensor-less FM, and other unexpected human factors may challenge the accuracy of the sensor-less FM. Some of these factors could be managed via tele-medicine and live interaction with patient (e.g., via zoom, video-connect, or other tele-conference resources). However, the accuracy of this solution needs to be validated in a follow-up study. To reduce the sensitivity to camera view for unsupervised applications, a potential solution could be the use of advanced image processing approach (e.g. camera stereo) to detect the 3D trajectory of the forearm movements from the video. Second, this study only examined the differences in physical frailty between COPD patients and healthy subjects in a cross-sectional trial and in a small sample size. In future, a longitudinal trial will be conducted to examine whether the sensor-less FM is sensitive to track the change in physical frailty over time and/or in response to an intervention. In addition, the ability of the sensor-less FM to distinguish frail from non-frail participants should be confirmed in a larger sample size.

In conclusion, this paper proposes a sensor-less FM based on a tablet camera and image processing technologies. The sensor-less FM extracts frailty phenotypes and frailty index from the video of the 20-second repetitive elbow flexion and extension test with good reliability using the sensor-based FM as a benchmark. The sensor-less FM addresses the lack of an objective tool to remotely assess physical frailty for COPD patients. Furthermore, it can be widely used in general older population who have limited access to the medical facilities and need remote tracking of physical frailty. Compared with the sensor-based FM, the widespread availability of image-acquisition tools such as smartphones or tablets would make the deployment of the sensor-less FM more practical and less resource intensive.

## Figures and Tables

**TABLE 1.** Frailty related features (digital biomarkers) used to quantify 20-second repetitive elbow flexion/extension test.

Frailty phenotype	Features	Definition
Slowness	Speed, deg/s	Elbow angular velocity range
	Rise time, s	Duration of flexion acceleration (Fig 3.a)
	Fall time, s	Duration of flexion deceleration
	Extension time, s	Duration of flexion (Fig 3.b)
	Flexion time, s	Duration of flexion (Fig 3.c)
	Flexion/Extension time, s	Total duration for a cycle of flexion and extension
	No. of Flexion/Extension, N	Number of repetitions per 20 seconds
Weakness	Power, deg^2^/s^3^	Product of the angular acceleration rang and the range of angular velocity
Rigidity	Range of motion, deg	Range of flexion/extension rotation
Exhaustion	Decline in speed, %	Difference between the first and last 10 seconds of angular velocity, estimated as percentage of average speed
	Decline in power, %	Difference between the first and last 10 seconds of power, estimated as percentage of average power
	Increase in flexion/extension time, %	Difference between the first and last 10 seconds of flexion/extension time, estimated as percentage of average flexion/extension time
	Increase in rise time, %	Difference between the first and last 10 seconds of rise time duration, estimated as percentage of average rise time
Unsteadiness	Speed variability, %	Coefficient of variation (CV) of speed
	Rise time variability, %	CV of rise time
	Flexion time variability, %	CV of flexion time
	Extension time variability	CV of extension time
	Flexion/Extension variability, %	CV of Flexion/Extension time
	Power variability, %	CV of power
	Rigidity variability, %	CV of rigidity

**TABLE 2.** Upper extremity frailty parameters for both sensor-based and sensor-less FMs.

	Phenotype	Sensor-based	Sensor-less	Correlation Coefficient
Rise Time, s	Slowness	215.16±62.89	212.2±61.2	0.99*
Power, deg^2^/s^3^, × 100,000	Weakness	246.2±122.7	272±146.3	0.91*
Range of motion, deg	Rigidity	111±17.6	110.6±19	0.74*
Decline in power, %	Exhaustion	−4.1±13.2	−3.3±25.85	0.63*
Decline in speed, %		−1.35±8	−0.35±13.95	0.72*
Elbow flexion time, ms		343.6±95.1	337.7±91.4	0.99*
CV of elbow flexion time, %		8±2	9±3	0.23
CV of elbow extension time, %		7±2	8±3	0.12
Frailty index, score		0.18±0.05	0.17±0.05	0.75*

*: correlation satisfied statistical significance. Deg: Degree; s: second, ms: millisecond

**TABLE 3.** Group comparison of frailty Phenotypes (slowness, weakness, rigidity, and exhaustion) and frailty index between COPD patients and healthy control subjects using sensor-less FM.

	Control	COPD	P-value*	d**
Slowness	173.6±33.5	251±58.9	0.005	1.62
Weakness	381±128.5	163.1±50.1	<0.001	2.24
Rigidity	113.1±15.9	108.8±19.8	0.242	0.23
Exhaustion	4.8±29.59	−11.0±21.1	0.387	0.39
Frailty index	0.14±0.03	0.25±0.05	0.007	1.70

* Results adjusted by age and BMI;

** Cohen’s effect size d
